# Enhancing group‐based internet obesity treatment: A pilot RCT comparing video and text‐based chat

**DOI:** 10.1002/osp4.371

**Published:** 2019-10-28

**Authors:** Delia S. West, M. Stansbury, R. A. Krukowski, J. Harvey

**Affiliations:** ^1^ Arnold School of Public Health University of South Carolina Columbia South Carolina; ^2^ Health Sciences Center University of Tennessee Memphis TN; ^3^ Department of Nutrition and Food Sciences University of Vermont Burlington VT

**Keywords:** behavioural weight control, online, videoconferencing, weight loss

## Abstract

**Objective:**

Internet delivery of behavioural weight control interventions offers potential for broad geographic reach and accessibility, but weight losses online fall short of those produced with the same programme delivered in‐person. This pilot study examined feasibility and preliminary efficacy of a video‐based platform for delivering weekly chat as part of a 6‐month, 24‐session online group behavioural weight control programme compared with the established text‐based format, which has produced the best online weight losses to date.

**Method:**

Women with obesity (N = 32) were randomized to either (a) weekly video group chat sessions and provided with a cellular‐enabled scale (Video) or (b) Text‐based weekly chat sessions and given a digital scale (Text) and followed for 6 months to determine weight loss and treatment engagement.

**Results:**

Women randomized to the ideo condition lost more weight than those in the Text condition (−5.0 ± 6.0% vs. −3.0 ± 4.1%, respectively) at 6 months, although the difference was not statistically significant. However, women in the Video condition had significantly greater treatment engagement, with greater self‐monitoring and website utilization than those in the Text condition.

**Conclusions:**

Videoconference delivery of group‐based online weight control accompanied by a cellular‐connected scale may promote greater treatment engagement and weight loss than text‐based chat. A larger, adequately powered study is warranted to determine which elements drive these enhanced treatment outcomes.

## 1 INTRODUCTION

Obesity presents a vexing public health challenge. Effective behavioural weight control treatments are available,^1^ and projections show these interventions could have a significant impact on overall population health if the interventions were more broadly accessible.^2^ Internet delivery of behavioural lifestyle interventions is an attractive option because of the potential for reach.^3^ Internet‐delivered group behavioural weight control programmes that incorporate synchronous text‐based group sessions (ie, “chats”) can produce an average of 5% weight loss, with almost a quarter of participants losing at least 10% of their body weight by 6 months.^4^, ^5^ These studies demonstrate the potential of online behavioural weight control interventions to produce clinically meaningful weight loss for individuals residing across disparate geographic regions^6^; however, weight losses achieved with online programmes are significantly smaller than those produced with the same programme delivered in‐person,^4^ and reviews of available literature conclude that in‐person interventions are more effective at producing weight loss than web‐based approaches.^7^ Methods to enhance online weight control programme outcomes are necessary to realize the full potential of digital approaches.

A central question in the efforts to develop a maximally effective online weight management approach is, “What critical elements differentiate in‐person weight control programmes from their online counterparts that might account for the greater weight losses achieved in‐person?” Two distinguishing features between in‐person and online programmes are the actual face‐to‐face time with the counsellor and other group members throughout the session, as well as the pre‐session weigh‐in. These elements are intended to foster accountability and social support.

Delivery of treatment in a face‐to‐face setting is the archetype of behavioural weight control and current gold standard format of delivery for behavioural obesity treatment, as exemplified by the Diabetes Prevention Program Lifestyle Balance^8^ and the Look AHEAD Intensive Lifestyle Interventions.^9^ The social support provided by others in the group is a well‐documented aspect contributing to the effectiveness of in‐person lifestyle interventions.^10^ Seeing fellow participants face‐to‐face may encourage social support for weight loss efforts, particularly between individuals unknown to one another at the start of group, and foster stronger connections within the group. This is consistent with observations that participants receiving online obesity treatment via synchronous text‐based group “chats” report lower perceived social support from fellow group members than participants receiving the same behavioural treatment in an in‐person group setting.^4^ However, synchronous text‐based group chats and real‐time interactions with counsellors appear to produce greater weight losses than programmes that offer social support only through asynchronous postings on bulletin boards or email correspondence.^3^ Collectively, these results bolster the proposition that social support offered face‐to‐face may contribute to the potency of in‐person weight control.

Some have argued that face‐to‐face social support is richer with verbal and nonverbal cues (eg, tone of voice and facial expressions) than that available in computer‐mediated social support.^11^ With recent advances in technology, online audiovisual social interaction is possible through the widespread usage and integration of high‐quality webcams on desktops, laptops, and mobile devices, as well as internet connections that support the transmission of visual images. Indeed, focus groups utilizing online audiovisual technologies achieved more interaction and richer data than text‐only meetings.^12^ Further, in‐person and audiovisually conducted meetings resulted in comparable interaction between participants, as demonstrated by individuals directing their comments to other participants (rather than just to the facilitator) and by responses that indicate agreement or divergence with the comments of another participant.^12^ Thus, online audiovisual group sessions may offer advantages for delivery of web‐based behavioural weight control.

Initial explorations of video‐based chats as a delivery platform for behavioural weight control interventions have been promising. A commercial programme demonstrated that self‐reported weight losses among participants who elected to participate in an online videoconference programme were similar to those who selected in‐person treatment.^13^ Comparable, clinically‐meaningful weight losses were also achieved among military personnel who completed a structured group‐based behavioural weight control programme delivered in‐person or by videoconferencing (6.2% and 5.3%, respectively).^14^ Additionally, there were fewer dropouts with videoconferencing delivery.^14^ This suggests that online groups with audiovisual interaction may be a promising approach. However, use of web‐based videoconferencing to deliver group sessions as part of a structured online behavioural weight control programme has not yet been examined rigorously using objectively measured weight loss outcomes and a randomized design.

The individual weigh‐in prior to the group meeting is another element of traditional in‐person, group‐based behavioural weight control programmes that distinguishes the approach from their web‐based counterparts. Individuals arrive a few minutes before the scheduled group session for their private weigh‐in, which not only enables treatment staff to obtain an objective body weight measurement and directly view the individual's weight trajectory, but also offers an opportunity for interaction, provision of support, reinforcement, and problem‐solving, as necessary. Individuals then convene in the group meeting room for additional interaction as other group members assemble. The accountability and informal social support provided during the pre‐session weigh‐in period likely enhance weight loss efforts.^10^, ^15^ Current conceptualizations of online, synchronous chat‐based programmes do not afford this pre‐session opportunity for informal interaction, but such opportunities could be made available for participants to “virtually” enter the group room and interact informally prior to the scheduled session start time. Additionally, electronic scales used on the day of the online group meeting to transmit an individual's weight to treatment staff can potentiate provision of real‐time feedback (eg, via email) on weight changes observed. Reinforcement, encouragement, and strategies (eg, problem solving) can be offered, mirroring the typical practice of in‐person programmes.

Collectively, utilizing videoconferencing group delivery platforms and incorporating a virtual weigh‐in and informal social support opportunities prior to group start may narrow the gap between weight loss outcomes achieved in‐person and those achieved with online delivery, effectively marrying the advantages of face‐to‐face contact and social support with the accessibility advantages offered by internet delivery. Therefore, the current pilot study sought to examine the feasibility and preliminary efficacy of a video‐based group chat format and cellular‐enabled scale within an online behavioural weight control programme in comparison with the established text‐based group chat delivery approach within the same online programme^4^, ^5^ using a randomized controlled design.

## 2 METHODS

### 2.1 Study design

This 6‐month randomized controlled pilot study was conducted between March and November 2018 at two clinical sites (South Carolina and Vermont) and randomly allocated participants to either (a) a 24‐week, group‐based online behavioural lifestyle programme with weekly synchronous video‐based chat sessions and a cellular‐enabled scale (Video) or (b) the same online group programme with weekly synchronous text‐based chat sessions (Text). Text‐based chat was selected as the comparator because it has the best weight loss outcomes for synchronous online group weight control published to date and has been utilized to produce weight loss for almost a decade.^3^, ^4^, ^5^ Outcome data were collected at baseline, 2 months, and 6 months. The study was approved by the Committee on Human Research in the Behavioral Sciences at the University of Vermont and the Institutional Review Board at the University of South Carolina and registered at http://clinicaltrials.gov (NCT03491293).

### 2.2 Participants

Women were recruited through word‐of‐mouth and targeted emails using available distribution lists (eg, worksites, professional organizations, and sororities). Potential volunteers entered an online recruitment portal that included a study overview and initial screening questionnaire. A phone interview was conducted to determine likely eligibility, followed by an in‐person orientation session at which informed consent was obtained. To be eligible, women had to be in good health, 18 years or older, and have a body mass index (BMI; kg/m^2^) between 25 and 50. Individuals were ineligible if they had a heart attack or stroke in the past 6 months, had a history of bariatric surgery, lost more than 10 pounds in the previous 6 months, were taking medications for weight loss, had a condition for which weight loss was contraindicated, or would be unavailable for follow‐up data collection. Access to a computer with Internet and a smartphone (compatible with self‐monitoring apps for dietary intake and step‐counting) were required. Participants completed a behavioural run‐in consisting of successfully logging into both the text and audiovisual group chat websites and completing an online food diary for 3 days. Participants also had to agree to create an account with http://myfitnesspal.com to monitor their diet and physical activity daily and allow counsellors to view their diaries (to guide feedback) by “friending” the programme. Finally, they had to agree to be randomized. After baseline data collection, individuals were randomized using a biased coin approach with race balanced across conditions.

### 2.3 Online behavioural weight control intervention

Participants in both conditions were offered the same 6‐month manualized online intervention adapted from in‐person, group‐based behavioural lifestyle programmes,^8^, ^9^ which we have previously demonstrated to be effective.^4^, ^5^ The intervention focused on restricting calorie intake and increasing physical activity using self‐management skills, such as self‐monitoring, goal setting, problem‐solving, and relapse prevention. Groups met online for 1 hour each week in a text‐based or video‐based synchronous chat session facilitated by an experienced behavioural weight control counsellor, who conducted the group chats for both conditions. Participants from each clinical site were in both conditions, but participants were only able to access their assigned group to maintain separation between conditions and build group cohesion and social support. Access to a condition‐specific, password‐protected, dynamic study website was provided to all participants. The website included lessons and activities that supplemented each chat and facilitated enactment of the featured behavioural strategy. A bulletin board for group communications, educational resources, healthy recipes, weekly weight loss tips, and updates on local physical activity events were also available. The website was updated regularly and provided the same information to both conditions.

The programme was goal‐based, recommended a 10% weight loss goal for all participants and prescribed an individualized calorie goal on the basis of initial weight, as well as a dietary fat goal corresponding to less than or equal to 25% of calories from fat. Participants were provided graded exercise goals that progressed to 200 min/week of moderate‐to‐vigorous physical activity and graded step goals that progressed to 10 000 steps/day. Individuals were instructed to monitor their steps using their smartphone or a physical activity tracker and record dietary intake, minutes of physical activity, and number of steps taken in MyFitnessPal daily. Group counsellors sent participants weekly emails with tailored feedback on the basis of their self‐monitoring. The same weight loss goal, dietary and physical activity prescriptions, behavioural strategies, and tailored feedback targets were implemented in both conditions of the study.

### 2.4 Video‐based chat plus cellular‐enabled scale condition (Video)

Participants randomized to the Video condition received the online behavioural weight control intervention and met weekly on the Zoom videoconferencing platform (Zoom Video Communications, Inc) by accepting an invitation offered 15 minutes before group start time. Once in the chatroom, tiled videos of each participant were seen in real‐time by others in the chatroom, and an individual's video tile enlarged when they spoke. Participants were also provided with a cellular‐enabled digital scale (BodyTrace, Inc, Palo Alto, California) prior to the first group chat and were instructed to weigh themselves daily. The scale automatically transmitted the weight measurement to the secure BodyTrace website via cellular connectivity where progress could be viewed by the participant and group counsellor, but not other group members.

### 2.5 Text‐based chat condition (Text)

Individuals in the Text condition received the online weight management programme and weekly text‐based group chat meeting, accessed on the Adobe Connect platform (Adobe Systems, Inc). Static pictures of all Text group members were visible at the top of the screen. All interactions occurred via typed comments, and the acceptability of typos and grammatical errors was underscored. For self‐weighing, individuals utilized their own personal scale or were provided with a digital bathroom scale, which did not transmit data; they were counselled to weigh themselves daily and instructed to record their weight on the study website on the day of the group prior to chatting.

### 2.6 Measures

All measures were obtained at baseline, 2, and 6 months unless otherwise indicated. Sociodemographic data were obtained by secure online questionnaire at baseline.

#### 2.6.1 Weight and adiposity

Weight was measured using a calibrated digital research scale (Tanita BWB‐800A). Height was obtained at baseline using a stadiometer. Weight change was calculated as kilograms lost and percent weight loss. BMI was calculated as weight (kg)/height (m^2^).

#### 2.6.2 Process measures

Intervention participation was assessed throughout the programme. Attendance at chats and number of days a participant self‐monitored their weight, dietary intake, and physical activity were recorded. Self‐reported physical activity was calculated as total minutes/week on the basis of the values entered daily in MyFitnessPal. Website utilization patterns were examined for both conditions.

#### 2.6.3 Social Support

Group social support and counsellor‐participant interaction were assessed in several ways. The Perceived Group Social Support Scale was used to measure perceived social support from fellow members of the participant's chat group.^16^ The scale includes 20 items and higher scores represent greater perceived support. The scale has been shown to have good internal consistency (0.90), test‐retest reliability (0.83), and predictive validity.^16^ The quality of the counsellor‐participant relationship was assessed with the bonding subscale of the Working Alliance Inventory (WAI).^17^ The subscale measures the degree to which an individual bonded with their counsellor and has been shown to have good convergent and divergent validity with an internal reliability estimate of 0.85.^18^ Individuals rate the frequency that they felt understood and allied with their counsellor on 12 items, each with a 7‐point scale; items are summed, with higher scores representing a more positive alliance with the group counsellor. In addition, a single 10‐point Likert item asked participants to rate the degree of support she felt from her group counsellor, with higher scores indicating greater perceived counsellor support. All social support measures were administered at 2 and 6 months.

### 2.7 Analyses

This pilot study was designed to examine feasibility and, therefore, a formal power analysis was not conducted. Weight loss, treatment engagement, and social support were examined to provide preliminary insights into the comparative efficacy of the two chat delivery platforms. Descriptive statistics were conducted for participant characteristics at baseline, as well as retention and process measures. Missing data for primary outcomes (weight losses at 2 and 6 months) were imputed with baseline observation carried forward for intent‐to‐treat (ITT) analyses, and groups were compared using independent samples *t* tests. Per‐protocol analyses were also conducted post hoc to examine weight loss outcomes among those who completed treatment. Given the exploratory nature of a pilot study, we present weight loss outcomes using both ITT and completer analyses. The nonparametric Mann‐Whitney‐Wilcoxon test was performed for group comparisons of process measures and social support due to non‐normal distributions. All analyses were conducted using SAS version 9.4 (SAS Institute: Cary, North Carolina). Statistical significance was defined as *P* < .05 (two‐tailed).

## 3 RESULTS

### 3.1 Participant characteristics

**Figure 1:**
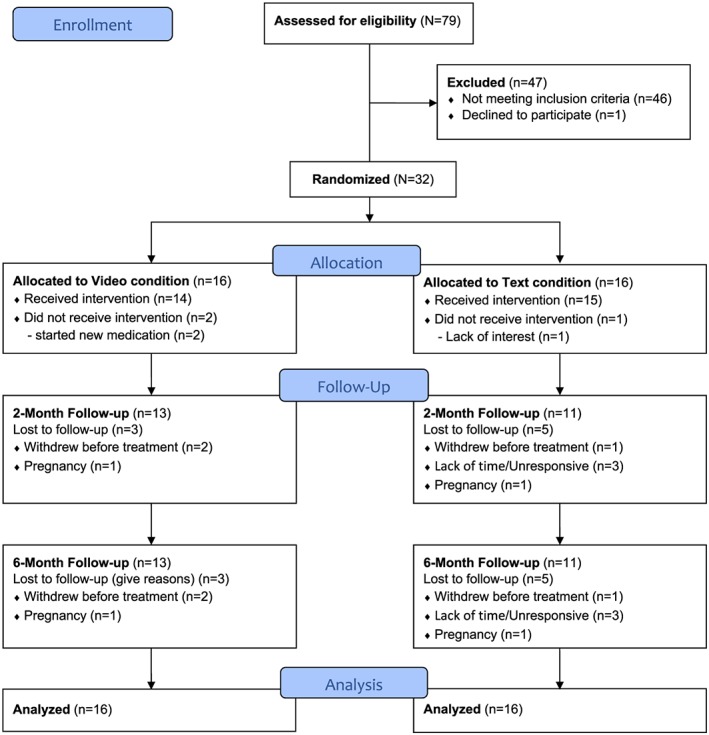
CONSORT flow diagram

Thirty‐two participants were randomized to the Video (n = 16) or Text (n = 16) conditions (Figure 1). On average, women were middle‐aged and well‐educated, with a mean BMI of 34.1 kg/m^2^ (69% had obesity), and 22% self‐identified as African American or another racial minority group (Table 1). There were no significant differences between conditions on baseline weight or other sociodemographic characteristics. Retention rates at 2 and 6 months were 78% and 75%, respectively, with no significant differences between conditions; however, the absolute number of individuals who withdrew early in the study in the Text group (31%) was higher than Video (12%).

**Table 1:** Baseline participant characteristics and retention rates

	Overall (N = 32)	Text (n = 16)	Video (n = 16)
Age (years)	47.2 ± 12.4	46.3 ± 12.3	48.1 ± 12.9
Race, n (%)			
White	25 (78.1)	12 (75.0)	13 (81.3)
African American	6 (18.8)	4 (25.0)	2 (12.5)
Other	1 (3.1)	0 (0.0)	1 (6.2)
Clinical site, n (%)			
South Carolina	16 (50.0)	8 (50.0)	8 (50.0)
Weight, kg	91.3 ± 14.0	93.5 ± 14.0	90.1 ± 14.3
BMI, kg/m^2^	34.1 ± 5.5	34.2 ± 5.4	34.2 ± 5.9
Obese (BMI ≥ 30), n (%)	22 (68.8)	11 (68.8)	11 (68.8)
Education, n (%)			
Some college (<4 years)	3 (9.0)	3 (19.0)	0 (0.0)
Vocational training post high school	1 (3.0)	1 (6.0)	0 (0.0)
College degree	14 (44.0)	7 (44.0)	7 (44.0)
Graduate/Professional	14 (44.0)	5 (31.0)	9 (56.0)
Retention rates			
2‐month follow‐up, n (%)	25 (78.1)	11 (68.8)	14 (87.5)
6‐month follow‐up, n (%)	24 (75.0)	11 (68.8)	13 (81.3)

*Note*. Values shown as mean ± SD or frequency counts (percentages).

### 3.2 Weight loss

On average, in the 2‐month ITT analyses, women in the Video group lost 2.7% compared with those in Text who averaged 2.2%, with no difference between the groups and no difference in the proportion of women achieving greater than or equal to 5% weight loss (Table 2). At 6 months, women in the Video group achieved an average weight loss of 2% greater than those in the Text group (5.0% vs. 3.0%, respectively), although this difference was not statistically significant. Further, there was no significant difference in the proportion of women losing greater than or equal to 5% or greater than or equal to 10% between the groups; however, twice as many women in Video have lost greater than or equal to 5% at 6 months compared with Text (six vs. three, respectively). An analysies of completers demonstrated the same pattern.

**Table 2:** Weight loss outcomes at 2 and 6 months

	Intent‐to‐Treat		Completers Only	
	Text	Video	Text	Video
2 months				
N included in analysis	16	16	11	14
Weight loss, kg	−2.1 ± 2.6	−2.3 ± 2.6	−3.0 ± 2.6	−2.7 ± 2.7
Weight loss, %	−2.2 ± 2.9	−2.7 ± 2.9	−3.2 ± 3.0	−3.1 ± 2.9
≥5% weight loss, n (%)	3 (18.8)	2 (12.5)	3 (27.3)	2 (14.3)
≥10% weight loss, n (%)	0 (0.0)	0 (0.0)	0 (0.0)	0 (0.0)
6 months				
N included in analysis	16	16	11	13
Weight loss, kg	−2.8 ± 3.8	−4.5 ± 5.6	−4.1 ± 4.0	−5.5 ± 5.7
Weight loss, %	−3.0 ± 4.1	−5.0 ± 6.0	−4.3 ± 4.3	−6.2 ± 6.1
≥5% weight loss, n (%)	3 (18.8)	6 (37.5)	3 (27.3)	6 (46.2)
≥10% weight loss, n (%)	2 (12.5)	2 (12.5)	2 (18.2)	2 (15.4)

*Note*. Values shown as mean ± SD or frequency counts (percentages) for intent‐to‐treat analyses using baseline observation carried forward for missing data. Independent samples *t* test for continuous variables. Fisher exact test for categorical variables.

### 3.3 Process measures

Engagement overall and by condition are shown in Table 3. Video participants attended an average of 15 (62%) chat sessions, and Text participants attended an average of 12 (50%), with no significant difference between groups. Similarly, daily self‐monitoring of dietary intake did not differ between groups. However, participants in the Video condition self‐monitored their weight on significantly more days (123 vs. 8 days) and reported physical activity significantly more often compared with those in the Text condition (55 vs. 22 days). No significant group differences were found for minutes per week of self‐reported physical activity; although, participants in Video reported substantially higher levels of physical activity (92 vs. 37 min). An analysis of only those who completed followed the same pattern (data not shown).

**Table 3:** Process measures for all randomized participants (ITT)

	Text (n = 16)	Video (n = 16)	*P* value
Attendance (out of 24 sessions)	11.9 ± 9.0	14.8 ± 8.6	.4057
Days of self‐monitoring (out of 161 days)			
Weight	7.7 ± 8.6	123.3 ± 63.7	<.0001*
Dietary intake	65.3 ± 55.4	101.0 ± 57.8	.0847
Physical activity	21.6 ± 20.7	55.1 ± 48.7	.0289*
Self‐reported physical activity (minutes/week)	36.6 ± 35.7	91.8 ± 107.4	.0919
Website Utilization			
Logins to website	22.8 ± 25.7	35.8 ± 37.9	.4275
Weekly lessons (out of 24)			
Lessons viewed	9.6 ± 8.4	16.6 ± 9.9	**.0463** *
Activities accessed	9.6 ± 8.3	15.7 ± 9.9	.0693
Activities submitted^a^	8.3 ± 7.7	13.9 ± 9.0	.0858
Discussion board use			
Times accessed	3.4 ± 4.7	12.1 ± 14.0	**.0165** *
Posts viewed	0.9 ± 1.5	8.2 ± 8.6	**.0010** *
Posts initiated	0.2 ± 0.4	2.7 ± 4.2	**.0297** *
Replies to posts	0.2 ± 0.4	2.9 ± 4.7	**.0293** *
Total interactions^b^	4.7 ± 6.0	25.8 ± 30.9	**.0125** *

*Note*. Values shown as mean ± SD.

a Activities submitted represents worksheets and other self‐paced lesson materials completed online and submitted for group counsellor review.

b Total interactions on Discussion Board aggregates number of times accessed, posts viewed, posts initiated, and replies to posts. Mann‐Whitney‐Wilcoxon test for all between‐group comparisons.

* Significant difference between conditions (*P* value < .05).

Website utilization demonstrated no significant difference between groups in the number of study website logins (Table 3). However, participants in the Video group were significantly more likely to view weekly posted lessons and access the discussion board, view posts, and initiate posts, suggesting greater overall engagement with the online programme.

### 3.4 Social support

Perceived group support was comparable between the two chat platforms, as was the counsellor‐participant bond and perceived support from the group counsellor (Table 4).

**Table 4:** Social support at 2 and 6 months

	2 months		6 months	
	Text (n = 13)	Video (n = 13)	Text (n = 8)	Video (n = 12)
Perceived group support	8.5 ± 3.5	10.7 ± 4.0	11.8 ± 2.9	11.0 ± 4.6
Counsellor support	7.5 ± 3.5	7.7 ± 3.0	8.5 ± 1.4	8.75 ± 1.6
Working alliance inventory				
Bonding subscale	67.5 ± 18.1	69.1 ± 12.8	76.3 ± 6.8	70.9 ± 13.1

*Note*. Perceived group support = Perceived Group Social Support Scale (range: 0 to 20). Counsellor support (range: 1 [not at all supportive] to 10 [extremely supportive]). Working Alliance Inventory Bonding Subscale (sum of 12 items per subscale; range per item: 1 [never] to 7 [always]). Values shown as mean ± SD. Mann‐Whitney‐Wilcoxon test for all between‐group comparisons.

## 4 DISCUSSION

Delivery of a 6‐month group‐based behavioural weight control programme utilizing a videoconferencing chat platform was feasible and effective. Weight losses in the Video group were greater than those achieved in the same programme delivered by text‐based chat (−5% vs. −3%, respectively), but the difference was not statistically significant, perhaps because of the small sample size in this pilot feasibility study. The observed effect size was 0.4, suggesting a moderate effect,^19^ which would be adequately powered to test differences in the means between groups with a sample size of 100 participants per condition. Participants in the Video condition were twice as likely to achieve clinically meaningful weight losses of greater than or equal to 5% as were those in Text (38% vs. 19%, respectively), but these differences also did not achieve statistical significance, again likely because of the small sample size. Thus, this pilot feasibility study demonstrated weight loss differences supporting the expected benefits of videoconferencing but did not reach the level of demonstrated efficacy that would be expected in a full‐scale trial.

Nonetheless, significantly greater engagement in the group‐based programme delivered with videoconferencing was demonstrated across several key domains. Those in the Video group self‐monitored their weight and physical activity on significantly more days than those in the Text condition. Self‐monitoring is consistently and robustly associated with better long‐term weight loss outcomes,^20^ and daily self‐weighing has emerged as a particularly effective behavioural strategy to promote weight loss.^21^, ^22^ Greater frequency of self‐weighing in the Video condition may have been facilitated by the provision of the cellular‐enabled scale, which transmitted weight data directly to intervention staff, while participants in the Text group had to enter their weight in the online self‐monitoring portal for a weigh‐in to “count.” However, participants in the Text group were provided with a digital scale if they did not have their own, so differences in access to body weight scales do not account for observed differences in self‐weighing practices.

Substantially greater website utilization was observed among those in the Video group. Women in the videoconferencing condition were significantly more likely to access lesson materials and to view and engage in discussions with their fellow group members on the bulletin board than those in the Text group. Further, Video participants logged into the website about 50% more often than Text participants. Engagement with the website has been shown to be strongly correlated with weight loss in other studies of online behavioural weight control.^23^

Surprisingly, perceived social support from group members and the counsellor was not higher among those meeting in the audiovisual forum than those receiving text‐only chat, despite the higher engagement in social support website features among those in Video. Comparable ratings of social support were observed in the two conditions, with participants in both Video and Text reporting relatively high levels of social support from their group and their counsellor. This may have resulted in a ceiling effect. Previous weight control studies using the same measure to characterize perceived group support for text‐based online and in‐person programmes have reported values of 6.4 to 7.9, respectively,^4^ compared with values of greater than 11 in the current study. Further exploration is warranted to determine whether increased treatment engagement and relatively higher weight losses among those experiencing audiovisual chat compared with those receiving text‐based chat are mediated by greater social support or by some other mechanism.

Retention differences in the two delivery channels did not rise to the level of statistical significance but were noteworthy in that 31% of women randomized to Text dropped out early on (ie, within the first 8 weeks). Only 12.5% of women in Video had dropped out at that point and one additional woman dropped out by the 6‐month assessment. Other quasi‐experimental studies have found greater drop out in the comparator arm relative to the videoconferencing arm,^14^ but substantial differences between that study design and the current study preclude drawing firm conclusions about superior retention when weight management is delivered via interactive video platforms.

Another puzzling observation is that weight losses achieved in the Text arm of this pilot feasibility study were smaller than produced in previous administrations of the same online, text‐based group behavioural weight control programme. In the current study, women in Text lost an average of 3% at 6 months; in previous studies, individuals receiving the text‐based chat condition averaged weight losses of greater than 5%.^4^, ^5^ Similarly, participants in the Text arm of the current study attended fewer chat sessions (50%) than did participants in previous trials of the text‐based programme who attended 66% to 76% of weekly chats.^4^, ^5^ It is unclear why these differences in engagement occurred. Previous studies were not limited to women, with approximately 10% men enrolled in the prior studies; men tend to lose more weight than women in the first year of a weight loss programme,^1^ and this may help explain observed differences. Another consideration is that online weight control is no longer novel. The earlier studies were implemented over a decade ago at a time when digital delivery of weight management was quite novel and “apps” for weight control were unavailable. The current study was implemented in the context of plentiful digital weight loss options in the marketplace, resulting in significantly reduced novelty for most participants. Further, weight losses achieved in the Text group (−2.8 kg) are consistent with those reported in recent evaluations of commercial online programmes (−2.7 kg),^24^ suggesting that weight losses of this magnitude may be becoming the norm as people become experienced (and less engaged) with digital platforms for weight management.

The study has several strengths worth noting. The randomized design and the objectively measured weight loss outcomes provide greater rigour than previous studies examining audiovisual chat as part of weight control. In addition, the same counsellor delivered both intervention groups, so differences between groups are unlikely to reflect interventionist skill or style. However, there are substantive limitations that merit discussion, foremost being the small sample size. The study was not powered to detect a 3% vs. 5% between‐groups difference in weight loss; rather, it was intended to examine the feasibility of this innovative approach to online delivery and generate data on which to power a full‐scale trial. Further, study results can be generalized only to women, and it is unclear how men might perform in text‐based versus video‐based delivery platforms. Finally, the video condition combined both audiovisual chats and provision of a cellar‐connected “smart” scale, and the impact of these two factors cannot be dissociated. Provision of a similar scale to individuals in an online commercial weight management programme has been shown to increase weight losses over a 6‐month period,^25^ and, therefore, may have contributed to the weight loss outcomes observed in the current study. The elements of the “treatment package” remain to be disentangled in future studies.

In conclusion, this pilot feasibility study suggests that videoconference delivery of group‐based chat and provision of a cellular‐connected scale within the context of a structured online behavioural weight control programme may promote greater engagement and more frequent self‐monitoring, as well as greater weight loss. A larger, adequately powered study to determine which elements drive these improvements in treatment engagement is warranted, as is exploration of the role of social support in video‐based chat, in efforts to merge the advantages of in‐person implementation of behavioural weight control with the accessibility advantages offered by internet delivery. Online behavioural weight control has such strong promise for overcoming geographic constraints, increasing dissemination of evidence‐based obesity treatment and reducing participant burden that continued investigation to refine internet‐delivered programmes to maximize weight losses and foster high levels of engagement is clearly warranted, and video delivery of interactive group sessions merits strong consideration as the process of refining online programmes progresses.

## CONFLICT OF INTEREST STATEMENT

No conflict of interest was declared
